# Current status of therapeutic monoclonal antibodies against SARS-CoV-2

**DOI:** 10.1371/journal.ppat.1009885

**Published:** 2021-09-03

**Authors:** Sanjeev Kumar, Anmol Chandele, Amit Sharma

**Affiliations:** 1 ICGEB-Emory Vaccine Center Program, International Centre for Genetic Engineering and Biotechnology, New Delhi, India; 2 Structural Parasitology Group, International Centre for Genetic Engineering and Biotechnology, New Delhi, India; 3 National Institute of Malaria Research, Dwarka, New Delhi, India; Mount Sinai School of Medicine, UNITED STATES

## Introduction

The ongoing Coronavirus Disease 2019 (COVID-19) pandemic has taken a toll on millions of lives worldwide. Currently, India has the second highest number of active COVID-19 cases and ranks third for the total number of deaths worldwide. While few vaccines are currently approved for use in India and elsewhere, there is still an urgent need for complementary approaches to tackle the current disease burden. Human monoclonal antibodies (mAbs) that neutralize Severe Acute Respiratory Syndrome Coronavirus 2 (SARS-CoV-2) and its variants provide an attractive treatment strategy. To this end, very recently, a formulation of human mAbs (casirivimab and imdevimab) against SARS-CoV-2 was approved for passive immunotherapy in mild and moderately severe COVID-19 cases in India and elsewhere. Thus, it is valuable and timely to summarize the specificity and reactivity of human mAbs against SARS-CoV-2 and its rapidly emerging variants.

### Primary targets of SARS-CoV-2 therapeutic neutralizing antibodies

**Fig 1:**
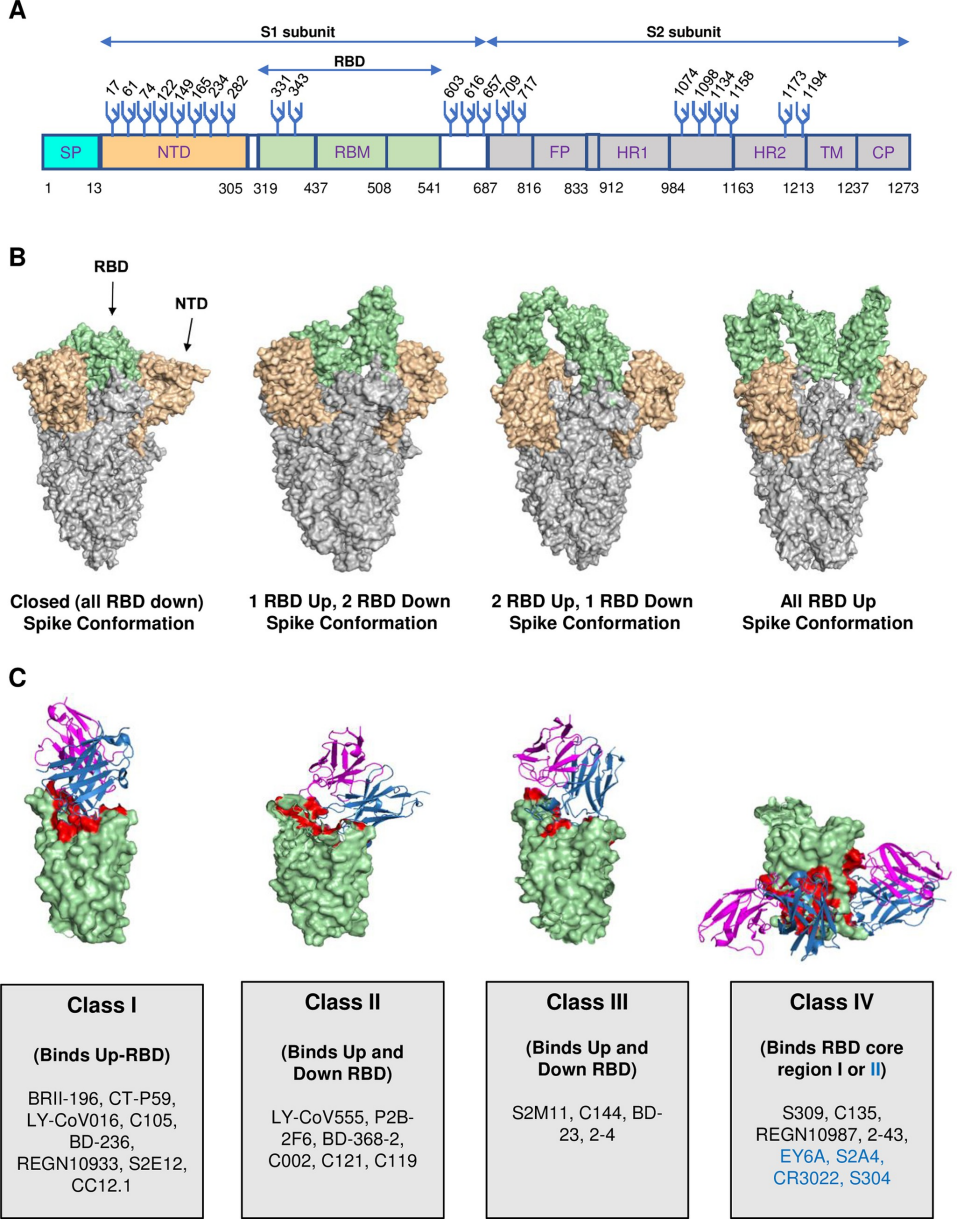
SARS-CoV-2 spike protein structure, conformation, and targets of RBD-dependent mAbs. **(A)** SARS-CoV-2 spike protein regions with amino acid position numbering are shown, which includes S1 domain regions: the SP, NTD, RBD, and RBM and S2 domain regions: FP, HR1 and HR2, TM, and CP. The glycosylation sites are numbered and marked with Y-like structures in blue. **(B)** Different conformations of spike protein (PDB: 7DF3, 6XKL, 7EB5, and 7KML, left to right). **(C)** Representation of 4 classes of SARS-CoV-2 RBD dependent mAbs. Antibody variable heavy chain region (sky blue) and light chain variable region (magenta) are marked. Antibody constant regions were removed from the bound Fab for clarity. The RBD is shown in (pale green) color and antibody contacts on RBD are marked in red (PDB ID: left to right, 7CM4 (CT-P59), 7CHF (BD-368-2), 7K90 (C144), and 6R6X (S304 (left) and S309 (right)). RBD class IV core II region targeting mAbs are shown in blue. CP, cytoplasmic tail; FP, fusion peptide; HR1, heptad repeat 1; HR2, heptad repeat 2; mAb, monoclonal antibody; NTD, N-terminal domain; PDB, Protein Data Bank; RBD, receptor-binding domain; RBM, receptor-binding motif; SARS-CoV-2, Severe Acute Respiratory Syndrome Coronavirus 2; SP, signal peptide; TM, transmembrane domain.

The spike (S) protein of SARS-CoV-2 is the primary target of neutralizing antibodies (NAbs) (**Fig 1A**). Therefore, NAbs against SARS-CoV-2 that have either been deployed for therapy or are in advanced stage trials, for the most part, either target the receptor-binding domain (RBD) or the N-terminal domain (NTD) of the spike glycoprotein (**Fig 1A and 1B**) [1,2]. The S protein exists in different conformations within the host, and their nomenclatures are based on the position of RBD protein—an “up” or “down” position (**Fig 1B**). To this end, based on the epitope recognition and binding mode, RBD-specific NAbs are categorized into 4 major classes (I, II, III, and IV) [3–5]. Class I and II NAbs bind the angiotensin converting enzyme 2 (ACE2) binding region or “receptor-binding motif” (RBM) region of the RBD on the spike glycoprotein [3]. The RBM region is responsible for the primary contact with host ACE2 to initiate the entry of the virus [6]. MAbs that block this RBM–ACE2 interaction are “ACE2 blockers.” The class I NAbs bind RBD in “up” conformation only and block ACE2 binding, whereas class II NAbs block ACE2 binding, and recognize both “up” and “down” RBDs (**Fig 1B and 1C**). On the other hand, the class III NAbs block ACE2 binding site, recognize spike protein with both “up” and “down” RBD conformations, and can interact with adjacent RBD protomers. The class IV NAbs do not overlap with ACE2 binding site and bind conserved region in RBD (core I region) or RBD in “up” conformation only (core II region) (**Fig 1B and 1C**). A complete description of these 4 classes of RBD-dependent mAbs is shown in **Fig 1C**. Class IV core I region-dependent NAbs have broad neutralizing activity against SARS-CoV-2, its variants, and other related coronaviruses [1–3,7]. Very recently, NAbs targeting new epitopes on the S2 domain (stem helix region) of spike have also been identified that are broadly neutralizing, i.e., neutralize SARS-related and other human coronaviruses (hCoVs) [8–11].

### Therapeutic COVID-19 mAbs in the clinic and in clinical trials

**Fig 2:**
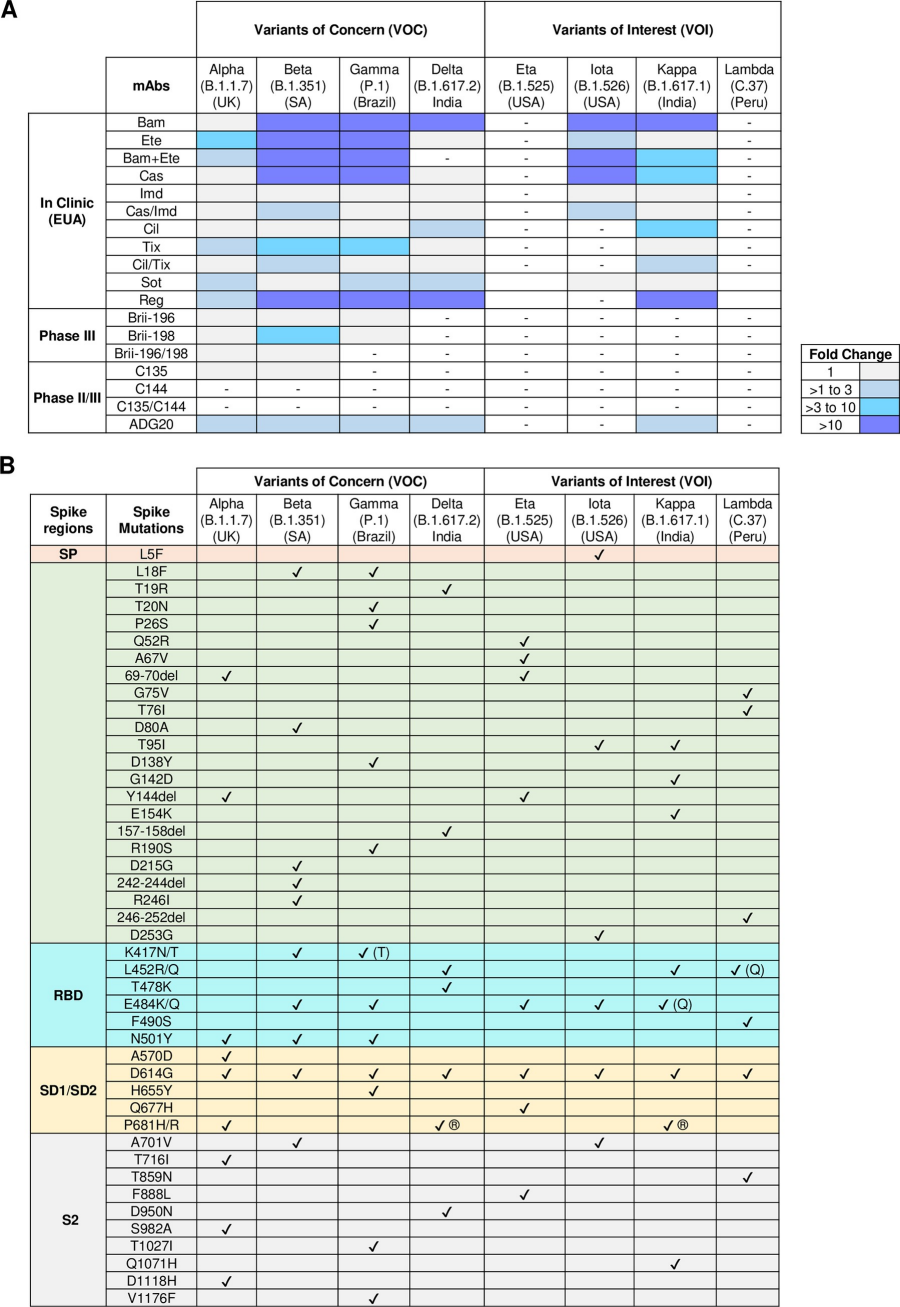
Neutralization potential of therapeutic mAbs against SARS-CoV-2 VOCs and VOIs. **(A)** Neutralization potential of SARS-CoV-2 mAbs at various stages of development/clinic against VOCs and VOIs. Here, fold change represents the reduction in IC50 values of SARS-CoV-2 variant neutralization in comparison to wild-types virus. The abbreviations for mAbs in the clinic (EUA) are the following: Bam, Bamlanivimab (LY-CoV555); Ete, Etesevimab (LY-CoV016 or JS016 or CB6); Bam/Ete, Bamlanivimab+Etesevimab; Cas, Casirivimab (REGN10933); Imd, Imdevimab (REGN10987); Cas/Imd, Casirivimab+imdevimab (REGN-COV2); Cil, Cilgavimab (COV2-2130 or AZD1061); Tix, Tixagevimab (COV2-2196 or AZD8895); Tix/Cil, Tixagevimab+Cilgavimab; Sot, Sotrovimab (Vir-7831 or S309); Reg, Regdanvimab (CT-P59). **(B)** List of mutations present in the current SARS-CoV-2 VOCs and VOIs. EUA, emergency use authorization; mAb, monoclonal antibody; RBD, receptor-binding domain; S2, S2 subunit; SARS-CoV-2, Severe Acute Respiratory Syndrome Coronavirus 2; SD1, subdomain 1; SD2, subdomain 2; SP, signal peptide; VOC, variant of concern; VOI, variant of interest.

Therapeutic mAbs for COVID-19 treatment have been developed in accelerated time and the pace has been unprecedented for any disease. The approvals were obtained in a record time of only 10 months, including 3 to 4 months of clinical grade mAbs production since the discovery of mAbs [12]. Currently, 8 SARS-CoV-2 RBD-specific potent NAbs have been approved by the Food and Drug Administration (FDA) under an emergency use authorization (EUA) to treat COVID-19 nonhospitalized patients at high risk of severe illness. The following COVID-19 mAbs are in clinical use: bamlanivimab (LY-CoV555) [13]; bamlanivimab (LY-CoV555) and etesevimab (LY-CoV016 or JS016) [14] from Eli Lilly; casirivimab (REGN10933) and Imdevimab (REGN10987) [15] from Regeneron; cilgavimab (COV2-2130 or AZD1061) and tixagevimab (COV2-2196 or AZD8955) [16] from AstraZeneca; monotherapy-based NAbs sotrovimab (VIR-7831) [17] from GSK and Vir Biotechnology; and regdanvimab (CT-P59) [18] from Celltrion. Another set of monotherapy and combination Nabs-based therapies are under Phase III trials: 2B04 [19] and 47D11 [20] from AbbVie; BRII-196 and BRII-198 from Brii Biosciences [2]; and TY027 from Tychan are also in Phase III trials [2]. A comprehensive list of NAbs that are currently in Phase I, II, and III trials and in clinic is summarized in **Fig 2A**. These therapeutic mAbs are used/administered in a range of 0.5 g to 1.2 g per dose, within 10 days of symptoms onset, as monotherapy or 2.4 g as a cocktail [21–23]. No dose-dependent effect was observed when these mAbs were tested at different doses (1.2 g to 8 g dose). The COVID-19 mAbs have demonstrated high efficacy in trials with a reduction of 70% to 85% in hospitalization or death [21,23,24]. Presently, these mAbs are used for intravenous administration; however, their intramuscular or subcutaneous administration testing is underway to facilitate larger access by overcoming the requirement of hospital settings. Presently, these mAbs are being produced in large-scale bioreactors of 15,000 L capacity, sufficient to provide 100 to 200,000 doses [12,25]. However, a single-dose regimen of these therapeutic mAbs continues to be expensive, particularly for low- and middle-income countries.

### What is the efficacy of SARS-CoV-2 mAbs against emerging variants?

Several SARS-CoV-2 variants are being reported from different parts of the world. According to the World Health Organization (WHO), a recognized mutation is elevated to a “variant of concern” (VOC) when the acquisition of a new mutation allows for increased viral transmission, increased fatality, and a significant decrease in the effectiveness of therapy and vaccines. A “variant of interest” (VOI) is a variant with a new mutation capable of affecting disease severity, transmissibility, immune and diagnostic escape. The current VOCs are Alpha (B.1.1.7, identified in the United Kingdom) [26], Beta (B.1.351, identified in South Africa) [27], Gamma (P.1, identified in Brazil) [28], and Delta (B.1.617.2, identified in India) [29]. The VOIs are Eta (B.1.525, identified in UK/Nigeria), Iota (B.1.526, identified in the United States of America) [30], Kappa (B.1.617.1, identified in India) [29], and Lambda (C.37, identified in Peru) [31] (**Fig 2B**). Recently, Epsilon (B.1.427/429, identified in the USA) [32], Zeta (P.2, identified in Brazil), and Theta (P.3, identified in the Philippines) [33] variants have been excluded from the category of VOIs by WHO due to their declining prevalence. Ideally, an effective antiviral therapeutic strategy should have the ability to prevent infection/disease by new variants while simultaneously maintaining breadth against existing multiple viral strains/variants. Recent studies have reported that many NTD-specific NAbs are relatively less effective to all emerging variants, whereas RBD-specific NAbs are variably effective against emerging variants and VOCs [2,34,35]. The majority of the potent therapeutic NAbs as monotherapy showed complete abrogation or reduced neutralizing activity against SARS-CoV-2 emerging variants that contain the E484K/Q or L452R mutations [34–37]. Bamlanivimab (LY-CoV555) was ineffective against all VOCs and thus was no longer considered for EUA. Currently, combination therapies comprising a cocktail of NAbs targeting distinct nonoverlapping epitopes on RBD have demonstrated exceptional potency and promising correlates of protection against SARS-CoV-2 and its variants (**Fig 2B**) [36,38]. Additionally, newly identified RBD core-binding NAbs SARS2-38 [39] and LY-CoV1404 [40] as monotherapy potently neutralize all SARS-CoV-2 VOCs. Therefore, several options of NAbs targeting conserved RBD epitopes are emerging as promising and attractive therapeutic candidates to tackle the disease burden caused by SARS-CoV-2 or its variants.

### What is the role of antibody Fc portion in therapeutic antibodies against SARS-CoV-2?

Although anti-viral functions of NAbs against rapidly emerging variants are being studied extensively, there is also a need to focus on understanding the role of the Fc portion of NAbs in providing protection against SARS-CoV-2 and emerging variants. The role of the Fc region is secondary when NAbs are administered as prophylaxis, but is critical for optimal therapeutic protection. A recent study demonstrated that therapeutic NAbs (REGN, Abbvie, AstraZeneca, and Vir Biotechnology) with intact Fc region reduced viral load and lung disease in animal models in comparison to NAbs without Fc effector functions (LALA-PG mutation) [41]. It is well appreciated that for optimal *in vivo* protection, NAbs with intact Fc region can mediate downstream effector functions via interaction with Fc receptors resulting in antibody-dependent cell-mediated cytotoxicity and antibody-dependent cellular phagocytosis. Moreover, Fc-mediated complement activation can exert a broad range of immunomodulatory functions, with activation of C1q resulting in antibody-mediated complement activation, and complement-dependent cytotoxicity. However, a recent study showed the diminished role of the Fc region in protecting against lethal SARS-CoV-2 infections in K18-hACE2 transgenic mice [42]. This study showed that potent NAbs do not rely on Fc effector functions to provide optimal protection when administered as therapy. Moreover, the role of Fc-mediated antibody-dependent enhancement (ADE) observed *in vitro* is yet to be fully elucidated in vivo [43,44].

### Benefits of multispecific antibodies

Recently, a few bispecific NAbs have been developed by combining the antibody chains of 2 independent nonoverlapping antibodies [45,46]. These bispecific NAbs neutralize wild-type SARS-CoV-2, its VOCs, and escape mutants and have shown to be protective in mice models. This suggests that bispecific NAbs are promising next-generation cost-effective therapeutics against SARS-CoV-2 and its VOCs. Such variant-resistant next-generation or combination of broadly reactive ultrapotent NAbs-based safe therapeutics are desperately needed globally. These mAb-based therapeutics should be globally accessible and affordable in low-middle income countries where more than of 85% human populations reside. Therefore, the development of a panel of well-characterized, clinically developable ultrapotent NAbs could be established rapidly to combat current and rapidly emerging SARS-CoV-2 variants.

## Conclusions

Here we have summarized the current status of mAb-based therapy for COVID-19 and have shed light on the ongoing development of mAbs-based therapeutics against emerging SARS-CoV-2 variants. Due to the potential of newly emerging SARS-CoV-2 variants in the future, vaccines will need to be constantly reassessed for their efficacy. The mAb biotherapeutics are a promising strategy for immediate treatment/prophylaxis or in situations where vaccines are less effective—such as in immunocompromised individuals, young, elderly, and vaccine-hesitant individuals. MAbs can also be rapidly tailored, selected, or mined towards new variants. For this, we need more intensive studies to track viral evolution, analyze the human antibody repertoire, identify and develop pan-coronavirus NAbs that target evolutionarily conserved epitopes. These efforts will enable rapid and dynamic reconfiguration of existing NAb cocktails to cull new surges that are driven by SARS-CoV-2 variants.
