# Correction: Current status of therapeutic monoclonal antibodies against SARS-CoV-2

**DOI:** 10.1371/journal.ppat.1010983

**Published:** 2022-11-21

**Authors:** Sanjeev Kumar, Anmol Chandele, Amit Sharma

The ORCID iD for the corresponding author, Amit Sharma, is incorrect. The correct ORCID iD is: 0000-0002-3305-0034 (https://orcid.org/0000-0002-3305-0034).
